# Tracing the origin of heterogeneity and symmetry breaking in the early mammalian embryo

**DOI:** 10.1038/s41467-018-04155-2

**Published:** 2018-05-08

**Authors:** Qi Chen, Junchao Shi, Yi Tao, Magdalena Zernicka-Goetz

**Affiliations:** 1 Department of Physiology and Cell Biology, University of Nevada, Reno School of Medicine, Reno, NV 89557 USA; 2 Center for Computational and Evolutionary Biology, Institute of Zoology, Chinese Academy of Sciences, 100101 Beijing, China; 3 Mammalian Development and Stem Cell Group, Department of Physiology, Development & Neuroscience, University of Cambridge, Downing Street, Cambridge, CB2 3EG UK

## Abstract

A fundamental question in developmental and stem cell biology concerns the origin and nature of signals that initiate asymmetry leading to pattern formation and self-organization. Instead of having prominent pre-patterning determinants as present in model organisms (worms, sea urchin, frog), we propose that the mammalian embryo takes advantage of more subtle cues such as compartmentalized intracellular reactions that generate micro-scale inhomogeneity, which is gradually amplified over several cellular generations to drive pattern formation while keeping developmental plasticity. It is therefore possible that by making use of compartmentalized information followed by its amplification, mammalian embryos would follow general principle of development found in other organisms in which the spatial cue is more robustly presented.

## Abstract (web summary)

What breaks symmetry in early mammalian embryonic development has been much questioned. Here, Magdalena Zernicka-Goetz and colleagues propose that compartmentalized intracellular reactions generate micro-scale inhomogeneity, which is amplified in the developing embryo, driving pattern formation.

## Introduction

**Fig. 1:**
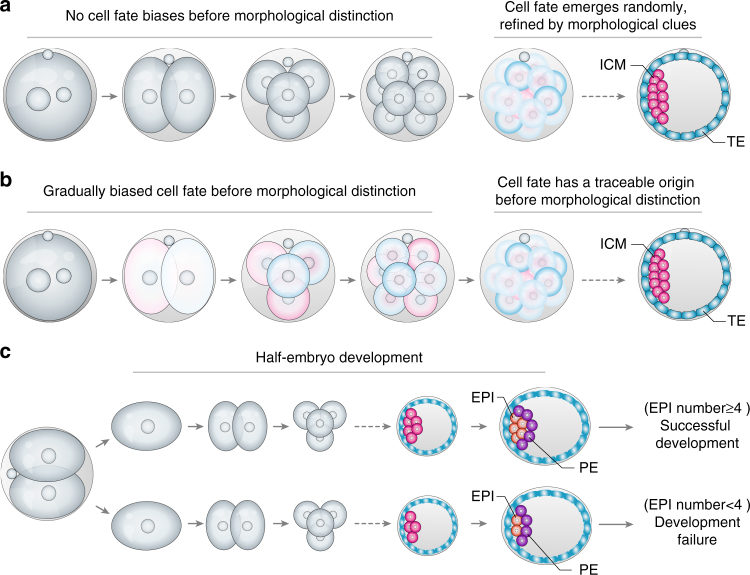
Different ideas of the first mammalian cell fate decision and clues from “half-embryo” development. **a**, **b** The timeline of mammalian embryonic development leading to specification of the embryonic inner cell mass (ICM) and extra-embryonic trophectoderm (TE) lineages, and the different views of the fundamental question of whether **a** the first cues for cell fate bifurcation in the mammalian embryo emerge randomly and then become refined by spatial cues effective after from the 16-cell stage onwards; or **b** whether molecular cues for differentiation emerge much earlier and guide cell fate specification by affecting cell position, cell polarity, and differentiation so finally cell fate. A fundamental question underlying these two different ideas is whether it is molecular cues that guide the morphological distinction, or the morphological distinction guides molecular clues toward cell fate decisions. What then, if both exist? **c** The chance of a “half-embryo” derived from a 2-cell blastomere developing into a mouse is not equal^15–19^. It depends on the number of epiblast cells generated by the embryo implantation^17^. EPI epiblast, PE primitive endoderm

How a single cell differentiates to have the distinct cell fates that give the blueprint of the new organism is a central question in developmental and stem cell biology. Although relevant to numerous model systems, this question has been particularly puzzling in mammalian embryos because their blastomeres appear to lack clearly visible pre-patterning determinants (i.e., morphogens), which are present in many other organisms^1^ (Box 1). And yet, on the third day after fertilization, two distinct cell lineages inevitably arise in the mouse embryo: the inner cell mass (ICM) that will generate the epiblast forming the new organism and the primitive endoderm forming the yolk sac, and the outside trophectoderm (TE) that will generate the placenta (Fig. 1a, b). The precise molecular trajectory of this bifurcation of fates, ICM vs. TE, has been difficult to track because until inside and outside cells form, all of the cells look identical and the embryo is developmentally plastic (Box 2). This has led to a long-lasting debate with two very different viewpoints of development of the early mammalian embryo. The first viewpoint argues that cell fate emerges randomly because an early embryo is homogeneous with all blastomeres identical to each other in their prospective fate and potential (Fig. 1a)^2–6^. The second viewpoint argues that cell fate can be predictable because an embryo is not perfectly homogeneous and consequently not all blastomeres identical, reflecting the differential expression and/or localization of molecules that drive cell character without restriction of developmental plasticity (Fig. 1b)^7–14^.

The first viewpoint represents the traditional way of thinking about mammalian development. The second viewpoint, although at first viewed with caution, is now gaining support as several studies have demonstrated inequality in the totipotency of blastomeres at the 2-cell and 4-cell stages of mouse embryos. It has been long known, for example, that when blastomeres are separated at the 2-cell stage, only one blastomere is able to develop into a mouse^15–19^. Such full developmental potential is only attained when the separated 2-cell stage blastomere generates sufficient epiblast cells by the blastocyst stage^15–17^ (Fig. 1c). These findings support the idea that 2-cell blastomeres do not have identical developmental potential. If cells of the classically studied mammalian embryo, the mouse embryo, indeed become different from each other already at the 2-cell stage of embryogenesis, how does this heterogeneity first arise? Can it be dormant and already present within the fertilized egg? If so, this would challenge the paradigm that the mammalian egg is homogenous, opening the question of what might break this homogeneity in the first place. Here we bring together new insights gained through the advances in single-cell transcriptome analysis^7,20–22^, in the quantitative imaging of live embryos permitting the tracking of cells and of molecules within them^9,11^, in mechanical analysis^23–26^, and in mathematical modeling^21^ to propose a new hypothesis. We propose that compartmentalized intracellular reactions generate micro-scale inhomogeneity, which is gradually amplified in the developing mammalian embryo. We propose that this drives pattern formation while retaining developmental plasticity.

### Box 1 Comparative view of embryo symmetry breaking in model organisms

Schematic depiction of early embryonic stages and cell fate specification in different model organisms (see Figure).

In worms, frogs, and sea urchins, the pre-patterning of the oocyte or zygote is very prominent with asymmetrically deposited morphogens (illustrated in different colors) that would dictate the fates of the descendent blastomeres. In contrast, mammalian embryos do not have an obvious asymmetric distribution of pre-patterning factors but, instead, develop and employ for cell fate specification more subtle clues (illustrated in different shades that become obvious at the 4-cell stage and which we discuss in detail in the perspective). Thus, while other embryos are viewed as developing in so-called “mosaic” fashion in which the perturbance of pre-patterning, for example by removing a blastomere that has the blueprint of a specific lineage, leads to a developmental deficiency, mammalian embryo development has the regulative nature in which the faulty development of a blastomere can be often, although not always, tolerated and embryo development proceed.

One explanation from an evolutionary perspective for why mammalian embryos adopt this unique strategy would be that mammals generate fewer eggs (around 1–20 per ovulation) than the other model metazoans depicted above (thousands to millions per spawning). As a result, the mammalian embryo might need a higher level of resilience to escape early embryo lethality. In fact, this plasticity has made it possible to perform pre-implantation genetic diagnosis^27^ by removing a blastomere from the embryo for genetic screening.

The developmental plasticity of the mouse embryo led to debate regarding their cellular and molecular strategies to achieve timely fate decisions as we discuss in this review (Fig. 1a,b). In our view, the lack of a robust deterministic pre-pattern does not mean that blastomeres are identical but rather than the differences between them are more subtle and gradually amplified to drive pattern formation while keeping developmental plasticity.
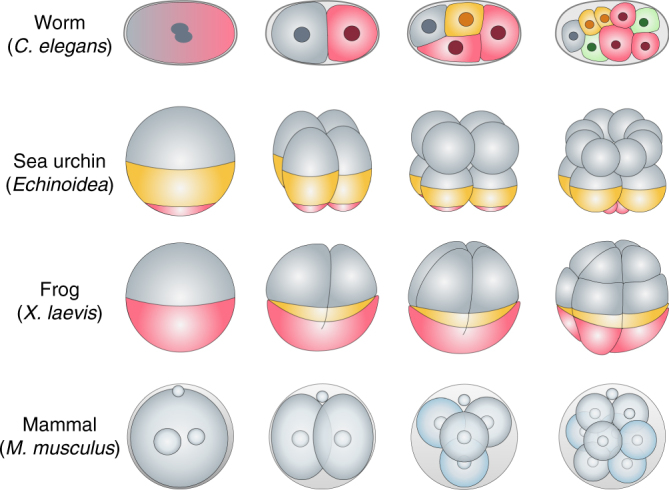


### Box 2 Stochastic and deterministic aspects of cell fate decisions

The regulatory vs. mosaic nature of cell fate specification process between embryos of mammals vs. other classic model metazoans (worms, sea urchins, and frogs) (Box 1) tends to raise the intuitive idea that the embryonic cell fate decision process in classic model animals is deterministic, whereas in mammals it occurs at random.

As depicted here, in a pinball model of bifurcation of fates (see Figure), the inside trajectory of classic model metazoans could be as deterministic as predesigned pinball tracks into fate A or B (see Figure, part a). However, does the plastic nature of embryonic cell fate in mammals mean that the trajectory is completely random and unpredictable as an unregulated bouncing pinball (see Figure, part b). In fact, predictable factors could be hardwired in a seemingly random system that inevitably bias the outcome. For example, when a “direction tube” (red) is embedded in the system, it redirects the trajectory of a moving pinball from any direction toward a defined fate. This will systematically change the probability of ending up in one fate (fate A) rather than another (fate B) (see Figure, part c).

When a more complicated system (which we call “The Game of Fate”) is embedded with multiple “direction tubes” with opposing effects (red vs. blue), and additional layers of regulators such as a “quality control bar”, a “reversal bar”, and “irreversible tunnels” as depicted (see Figure, part d), the system’s outcome in biasing the pinball’s fate would depend upon multiple factors such as the initial velocity, the angle of entrance, the number and relative positions of hardwired elements, and thus the orders in which they are hit/triggered during the movements. Despite a certain degree of inherent random behavior of the system, the triggering of defined regulatory elements can create a biased outcome. This Game could be to some extent analogous to a mouse blastomere with undecided fate, where lineage specifiers with opposing effects co-exist inside the blastomere along with other regulatory factors (e.g., cell position and cell contact) and so blastomeres remain in an intermediate state before their fate is defined^21^.
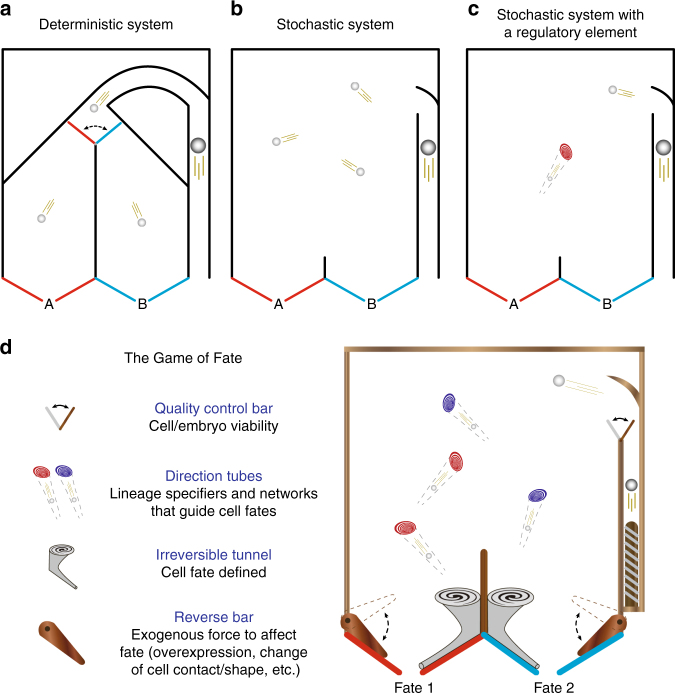


### Previous theories of biological pattern formation

The complexity of biological pattern formation has attracted not only biologists, but also physicists and mathematicians from the times of the philosopher Immanuel Kant (1790) who defined life as an emergence of functions by a “self-organized, self-producing” process^28^. Despite this great interest, the molecular and physical basis of how symmetry breaking and self-organization is initiated, at both cellular and organismal levels, remains not fully understood. We believe that the development of our understanding of biological pattern formation owes a great deal to Alan Turing’s seminal work “The chemical basis of morphogenesis”^29^. Here he proposed the idea of “morphogens”—chemical substances that interact with each other and diffuse in the system to define morphogenesis and pattern formation. Below we would like to take Turing’s idea further, and hypothesize that each of the essential elements of biological pattern formation such as gene expression, external forces, shape, motion can influence the generation or diffusion of morphogens leading to embryo pattering.

#### Alan Turing’s reaction-diffusion theory

**Fig. 2:**
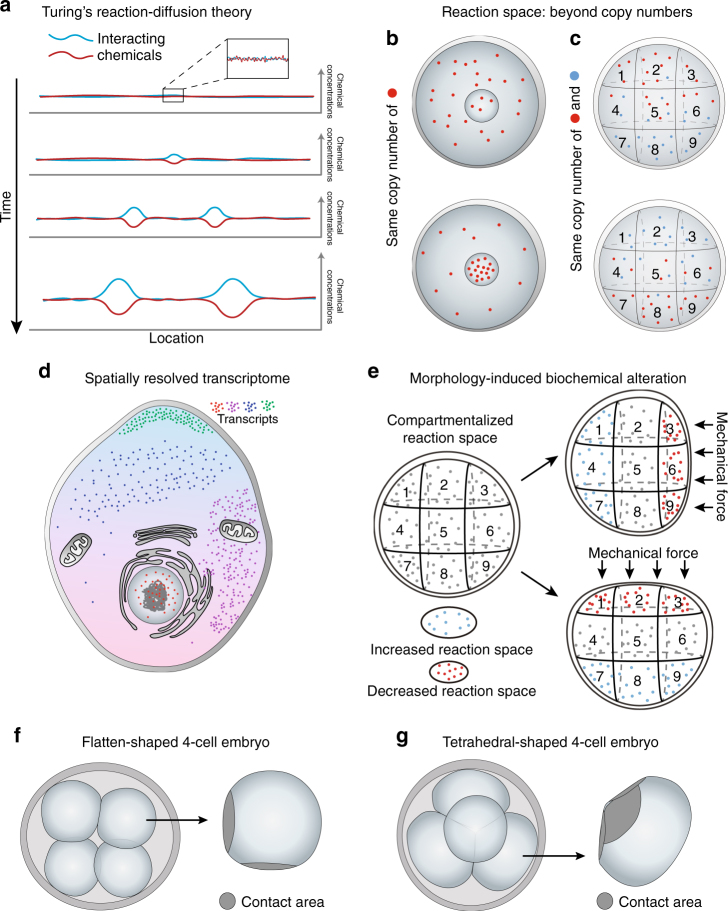
Origin of cellular pattern formation from Turing’s theory to compartmentalized intracellular reactions. **a** Turing’s reaction-diffusion theory illustrates how two interacting chemicals with different diffusion rates can generate stable heterogeneity from a homogenous system. **b**, **c** Without changing the number of molecules in a biochemical reaction, the spatial information where **b** a specific type of molecule resides in the system, or **c** the relative sub-location of multiple molecules can lead to differential outcomes/products, thus altering the features of the system. **d** Illustration of the spatial mapping of the transcriptome at a single-cell and sub-cellular level by emerging technologies. **e** The morphological changes of a cell, due either to cell–cell contact or external forces, results in an altered reaction space inside the cell, leading to region-specific changes in biochemical reaction rates and cell properties. **f**, **g** The 4-cell mammalian embryo can be either **f** flattened or **g** tetrahedral in shape; the cell geometry and contact areas of tetrahedral 4-cell blastomere differ from the flattened blastomeres, which may trigger differential changes in intracellular reaction space related to cell fate

Alan Turing was the first to employ mathematical modeling to illustrate how two interacting chemicals with different diffusion rates can generate stable heterogeneity out of a homogenous system, in what was to become the reaction-diffusion theory^29^ (Fig. 2a, Box 3). Turing’s model, which now has both chemical and biological experimental support^30–32^, provides a theoretical explanation for the establishment of distinct cellular compartments that differ first chemically and then morphologically. This process relies on both the effects of local self-enhancement and long ranging inhibition, relating to both the intrinsic properties of the chemical, as well as its positional information. From a modern biological perspective, Turing’s hypothetical chemicals and their reaction-diffusion properties could be considered as expressed gene products such as RNA, proteins, and gene regulatory networks, whereas the effects of local self-enhancement and long ranging inhibition could represent the source of spatial heterogeneity within the intracellular environment to result in differences between cells already at the 2-cell stage. It can therefore be reasoned that even small perturbations in the levels of transcripts or proteins resulting from differential inheritance during cell division, intrinsic gene expression noise, or variation in an external signal (e.g., intrusion of the sperm) have the potential to be amplified depending upon the intrinsic properties of the molecules (RNA, protein, chemical metabolite) and then transformed into defined signaling networks leading to biological pattern formation.

#### Physical and physiological cues in controlling active diffusion

In Turing’s model, the diffusion process could be initiated by random molecular movement (i.e., Brownian movement), a passive form of transport. However, in a real cellular or embryonic system, various physical and physiological mechanisms are likely to actively regulate the speed of diffusion resulting in versatile forms of patterning.

In a classic example of embryo symmetry breaking, the *C*. *elegans* zygote, the sperm-donated centrosome triggers a cortical flow, which is controlled by cytoplasmic streaming. This triggers the flow of the protein PAR (polarity protein partitioning-defective) into a reaction-diffusion system resulting in the asymmetric anterior–posterior distribution of two mutually excluding PAR proteins in the zygote^31^. Recently established non-invasive focused-light-induced cytoplasmic streaming confirmed that physiological manipulation of intracellular flow can translocate or even reverse PAR polarization^35^ highlighting the paramount importance of physical and physiological intracellular transport in pattern formation.

Whether the intrusion of sperm can also trigger an asymmetric distribution of molecules in the mammalian zygote is currently thought unlikely, because the mammalian zygote is conventionally believed to be homogenous and so lacking any asymmetry. But if this dogma is challenged and the mammalian embryo is not actually homogenous, is it possible that sperm triggers asymmetry also in the mammalian zygote but that it is beyond our current detection until amplified? The amplifying factors could be specific spindle orientation affecting the pattern of embryonic cleavage. We discuss these ideas below in the light of recent evidence.

### Box 3 Reaction-diffusion and reaction space

*Turing’s reaction-diffusion*

The central idea in Alan Turing’s “reaction-diffusion system” is that a homogenous system can develop a pattern or structure based on an instability of the homogeneous equilibrium without an initial asymmetry. The general two-component reaction-diffusion equations of chemical *A* and *B* are listed:

$$\frac{{\partial A}}{{\partial t}} = f\left( {A,B} \right) - \mu \left( {A,B} \right) + D_A\nabla ^2A$$

$$\frac{{\partial B}}{{\partial t}} = g\left( {A,B} \right) - \nu \left( {A,B} \right) + D_B\nabla ^2B,$$

where $\frac{{\partial A}}{{\partial t}}$ and $\frac{{\partial B}}{{\partial t}}$ are the concentration change rate of *A* and *B*, *f*(*A*, *B*) is the production function, and *μ*(*A*, *B*) is the degradation function of *A*; *g*(*A*, *B*) is the production function, and *v*(*A*, *B*) is the degradation function of *B*. The production and degradation function are together called the reaction process. $D_A\nabla ^2A$ and $D_B\nabla ^2B$ are the diffusion function of *A* and *B*. $D_A\nabla ^2A$ and $D_B\nabla ^2B$ follow Laplacian functions which give the difference between the concentration of target area and the average concentration of nearby areas. The reaction-diffusion system was supposed to be initially in a stable homogeneous condition, but disturbed slightly from this state by stochastic interferences (e.g., Brownian movement) that ultimately act to bring the system out of the stable state.

Turing’s original model is more focused on the reaction-diffusion process among cells while the reaction-diffusion system is also applicable for the concentration change inside one cell, where it generates micro-scale asymmetry of molecular gradients that can be harnessed for symmetry breaking by differential reaction space as introduced below.

*Reaction space beyond copy numbers*

In chemical and biochemical reactions, reaction space refers to the spatial information (volume, shape, etc.) where the molecular reactions take place. A master equation^33^ is created to describe the state of the system regarding the time evolution of the probability density function regarding the copy numbers of the molecular species participating in the molecular system. However, if the master equation possesses nonlinear transition rates, it may be impossible to solve it analytically^33^. Under this background, the parameter *Ω*, as introduced by N.G. Van Kampen^33^, can reflect the size and system space in many cases, and thus can be used to solve the master equation.

By introducing *Ω*, the master equation can be described by a more compact equation^34^ in which *Ω* represents the size of the system.

$$\frac{{{\mathrm {d}}P({\boldsymbol{X}},t)}}{{{\mathrm {d}}t}} = \Omega \mathop {\sum }\limits_{j = 1}^R \left( {\mathop {\prod }\limits_{i = 1}^N E^{ \pm S_{ij}} - j} \right)\tilde f_j\left( {{\boldsymbol{x}},\Omega } \right)P\left( {{\boldsymbol{X}},t} \right),$$

where *P*(***X***, *t*) is the probability distribution for the molecule number vector ***X***. *Ω* represents the size of the system in which biochemical reaction take place, where different types of chemical components, *N*, and number of biochemical reactions, *R*, are involved. *S* is the stoichiometric matrix *N* × *R* of the reaction network in which element *S*_*ij*_ indicates the stoichiometric coefficient for *i*^th^ chemical component in reaction *j*. The $E^{ \pm S_{ij}}$ is a step operator that removes *S*_*ij*_ molecules from *X*_*i*_ in the function of *f*(***X***_1_,…,***X***_*i*_…,***X***_n_). For example, $E^{ \pm S_{ij}}f({\boldsymbol{X}}_1,...,{\boldsymbol{X}}_i,...,{\boldsymbol{X}}_n) = f({\boldsymbol{X}}_1,...,{\boldsymbol{X}}_i \pm S_{ij},...,{\boldsymbol{X}}_n)$. ***x*** = ***X***/*Ω* is the stochastic concentration vector that reflects the fluctuation in the system. $\tilde f_j$ is the reaction rate of *j* with the given state ***x*** and system size *Ω*.

As a result, the system’s reaction rate can be altered simply by the fluctuation of the system size *Ω*, without changing the number of molecule *X*. In this regard, the spatial information where a specific type of molecule resides in a biochemical reaction system (Fig. 2b) or the relative sub-location of multiple molecules (Fig. 2c) is of paramount importance to the product outcome, and it may generate completely different features of the system.

### The origins of bias in mammalian embryo and how to amplify them

Recent advances in single-blastomere transcriptomic analyses in both mouse and human embryos have revealed small, but unexpectedly significant, differences between the blastomeres emerging immediately after the first cleavage division of the egg, thus at the 2-cell stage^20,21^. In theory, such small differences might be either neutralized, through the emergence of a “monostable” pattern, or amplified in a “bistable” pattern upon zygotic genome activation^21^. Advances in the tracking of cells and of molecules in living embryos combined with functional analyses have indicated that the second possibility is likely. It was found that differential behavior of epigenetic modifiers, such as the histone methyltransferase CARM1, and transcription factors, such as Sox21, between blastomeres of the 4-cell stage embryos acts to initiate cell fate specification^7,10,36^. Furthermore, it was found that the length of time a pluripotency transcription factor Sox2 remains bound to DNA is also significantly heterogeneous at the 4-cell stage and that this acts downstream of CARM1 activity^7,11^. Together, these new insights indicate that blastomere-to-blastomere differences in CARM1 activity established by the 4-cell stage lead to differential expression of targets of Sox2, such as Sox21 but also others. This can drive cells fate as that higher expression of Sox21 leads to lower expression of a transcription factor Cdx2, which induces differentiation. Lower Cdx2 expression, in turn, leads to diminished expression of polarity markers that establish apical domain^37^. And in turn, cells with a smaller apical domain are internalized and become ICM^7^. This example demonstrates how cell behavior (polarity and movement) regulating cell fate decisions beyond 8-cell stage can be, in fact, profoundly influenced by molecules inherited from previous division and so having a traceable origin.

While growing evidence supports existence of important cell fate directing differences between blastomeres in the mouse embryo, the origin of this early heterogeneity is still unclear. Since the potential influence of stochastic events (gene expression noise and uneven random segregation at cell division)^21,22^ is thoroughly reviewed elsewhere^38–41^, we feel it is equally important to explore an alternative hypothesis, namely, that cell-to-cell heterogeneity is initiated by pre-existing molecular inhomogeneity regulated by intracellular compartmentalization. Below we will discuss how such micro-scale inhomogeneity of the mammalian embryo may generate bias that when amplified is strong enough to shape cell fate.

#### Compartmentalized intracellular reactions

It is necessary for a cell to carry out its correct functions that it maintains its intracellular biochemical reactions in differentially sequestered reaction spaces. In some cases, these compartmentalized reactions are membrane bound to form specific subcellular organelles. For example, nuclear compartmentalization is essential to buffer transcriptional noise preventing random fluctuations in the cytoplasmic transcript abundance, contributing to controlled cell-to-cell variability^42–44^. Asymmetric distribution of organelles, such as mitochondria^45^, peroxisomes^46^, or germline cysts^47^, is now shown to effect the fate of daughter cells in mammary stem-like cells, epidermal stem-like progenitors, and maturing oocytes, demonstrating that compartmentalized intracellular reactions within or associated with organelles could regulate cell-to-cell heterogeneity and cell fate decisions. In addition, non-membrane-bound organelles, such as the centrosome or a large variety of nuclear speckles, also provide compartmentalized spaces. The association of centrosomes with polar granules in *Drosophila* offers one example of how spatial determinants may be captured by cytoplasmic organelles^48^ and nuclear speckles have been proposed as a system for the phase partitioning of proteins and transcripts within the nucleus^49^.

In addition to organelles, compartmentalized reaction space can generate gradients of biochemical products that become differentially distributed in the cell creating distinct cellular states and fates (Fig. 2b, c, Box 3). These can be represented as classical pre-determinants, such as the morphogens that are asymmetrically deposited in the eggs of worms, sea urchins, and amphibians (Box 1). In the mammalian zygote, where morphologically obvious asymmetry is lacking, developmental cues may have subtler forms. Such developmental cues might be the mechanical effects of sperm entry^50^ or the consequences of the previous asymmetric meiotic divisions during oocyte maturation^51,52^ leading to actin-based directional cytoplasmic streaming^53^ or localization of specific RNAs and/or proteins within spatially distinct intracellular microenvironments. Any or all of these factors might provide sources of initial heterogeneity that could be amplified and bias the cell division pattern and cell fate under Turing’s reaction-diffusion theory (Fig. 2a).

In agreement with this hypothesis, the cataloging of multiple transcripts within a cell using multiplexed subcellular RNA sequencing^54^ and FISH labeling^55,56^ revealed that the intracellular transcriptome is spatially compartmentalized (Fig. 2d). These observations stand alongside hypotheses that biochemical reactions within a cell are both confined and dynamically regulated in time and space^42,57^ and so might also serve as a source of intracellular symmetry breaking. We will next discuss these findings within the context of the early mammalian embryo.

#### Starting with bias

Recent studies revealed that subcortical maternal complex (SCMC) becomes localized asymmetrically during mouse and human embryogenesis^58,59^. It becomes excluded from cell–cell contact regions segregating to the outer cells from the morula stage onwards^58^. Genetic disruption of one the SCMC complex members, Tel6, results in the mis-localization of other SCMC members (MATER, Filia, and FLOPED) from the subcortical region leading to developmental arrest at the 2-cell stage^60^. These results support a hypothesis that the mammalian embryo always thought to lack asymmetrically distributed transcripts or proteins might actually possess gene products compartmentalized in different micro-environments, rather than being completely homogeneous. This initial asymmetry might be enhanced by biochemical or mechanical events at fertilization. This could be in the form of sperm-donated factors^61^ such as small RNAs, proteins, and organelles^62–64^ or through the mechanical impact inducing changes to egg shape in an actin-mediated process^65^. In support of this second possibility, it was reported that the mouse egg becomes flattened upon fertilization and that this influences spindle orientation and so how the zygote divides^65^. Moreover, removing and ectopically transplanting the opposite poles of the mouse zygote indicated that factors in the animal, but not the vegetal, pole contain positional information that determines the plane of zygote division^52^. In fact, the SCMC was also shown to be required for spindle positioning as deletion of its components results in an asymmetric zygotic division and abnormal distribution of organelles^60,66^. Mechanistically, the SCMC seems to control formation of the cytoplasmic F-actin meshwork and of cytoplasmic lattices as well as microtubule dynamics^60,66^, processes that all closely influence basic cellular architecture and that are potentially able to contribute to compartmentalizing reaction spaces in the mammalian oocyte and zygote. The defects in SCMC lead to reduced fecundity^59^ that could, at least in part, be due to an asymmetric distribution of critical molecules in 2-cell stage blastomeres. If so, this might link the biased determination of lineage fate with embryo lethality. Together, these findings support a hypothesis that spindle positioning in the zygote is influenced by pre-deposited micro-scale maternal factors, which, if disturbed, will generate embryo asymmetry and differential inheritance of subcellular compartments. This also implies the consequence of compartmentalized information in the mammalian embryo, which when amplified, could be as effective for patterning as the more robust determinants of other systems. It is therefore possible that by making use of initially compartmentalized information followed by its amplification, the mammalian embryo would ultimately follow a general principle of development found in other organisms in which the spatial cue is more robustly provided.

#### Mechanical and biochemical signal conversion

The evidence for a spatially confined subcellular transcriptome together with the idea of compartmentalized reaction space also provides a viable explanation of how mechanical cues, for example, cell–cell contact during development, can alter transcriptional patterns and cell fate^25,26^. In general, within a compartmentalized intracellular reaction space, the property of the space itself (volume and shape) has an essential influence on reaction rate^42,43^ (Box 3). Morphological changes to a cell, either due to intrinsic factors such as physical constraints arising during development or due to external forces, could alter the reaction space (Fig. 2e). This would then lead to region-specific changes in biochemical reaction rates and products that could alter intracellular properties. An example would be the molecular framework that triggers de novo cell polarization at the 8-cell stage^67^, which in turn may bias cell fate. In fact, the establishment of radial polarity by cell–cell contact and then the asymmetric trafficking of essential polarity RNAs/proteins is an essential step and generic principle in generating cell fate determination in multiple species, although the exact upstream mechanisms differ from case to case^68^.

It is possible to hypothesize that different cleavage division patterns characteristic of the mammalian embryo can result in differential cell–cell contact that affects blastomere geometry. This could create different reaction spaces even before cell polarization. The 4-cell embryo in the mouse and human, for example, is either tetrahedral or flattened in shape, depending upon a specific pattern of cleavage division (Fig. 2f, g), with the tetrahedral embryos showing a significantly higher developmental potential than the flattened ones^69–71^. Among other potential causes for this increased developmental potential is the expectation that each blastomere in tetrahedral 4-cell embryos will differ from those in the flattened embryos with respect to adhesive and tensional properties between cells (Fig. 2f, g). Such changes in cell geometry could trigger changes in the intracellular reaction space, leading to differential expression of molecular markers, organelle rearrangements and/or spindle orientation^72^, actin filaments and nuclear shape^73^. Any of these factors could profoundly affect cell fate and embryo quality.

Moreover, the mechanical forces of cortical tension and the autonomous contractility of blastomeres have been identified as factors that can modify cell position influencing cell fate^25,26^. During asymmetric divisions at the 8–16-cell stage, the more contractile blastomeres tend to move inward to become the ICM, whereas the less contractile blastomeres, which inherit more apical proteins, can induce nuclear accumulation of YAP and activation of the Hippo pathway, to become TE^25^. This supports findings that the initiation of Hippo signaling is controlled by cell polarity, prior to cell position^74–76^. Thus, polar and apolar cells pre-establish outer or inner features by differentially regulating Hippo signaling to activate downstream lineage-specific genes^77^. In this model, the mechanisms that establish the intrinsic differences between polar and apolar cells may be related to blastomere-to-blastomere contact and cell constraints during development, triggered by region-specific changes in biochemical reaction spaces; or facilitated by intercellular transport of essential molecules^78^. In addition, changes in cytoskeletal features can be directly relayed to the perinuclear/nuclear membrane structure, thus altering the state of the chromatin and influencing lineage-specific gene expression, as in other cellular systems^79^.

#### From subtlety to fate

Single-cell transcriptomic analyses of the mouse embryo revealed that blastomere-to-blastomere differences emerge already at the first cleavage division^21,22^. A stochastic aspect of this process could be due to a binomially distributed pattern of random segregation events, subject to physical laws with lower-quantity substances bearing a greater chance of being asymmetrically distributed^21^. But the initial transcriptome heterogeneity between blastomeres at the 2-cell stage may also result from compartmentalization of the transcriptome before the first cleavage division. In either case, any such small differences between blastomeres could trigger more significant asymmetries. In our view, this represents an important step in resolving the dilemma of how blastomeres of the 2-cell stage embryo can be both “identical” (they both contribute to the epiblast, although unequally) and yet “different” (with intrinsic heterogeneity that may guide cell fate) as is suggested by observed inequalities in totipotency at the 2-cell stage^15,80,81^. In a cell such as the fertilized egg, substances found in greater quantities are usually required for maintaining basic cell properties and these are encoded by the so-called housekeeping genes. In contrast, other essential regulatory elements, transcription factors or non-coding RNAs, are usually in short supply (as quantitatively defined in intestinal stem cells^82^) and have functions that are copy-number sensitive^38,83^. Therefore, if such essential regulatory factors are unequally distributed between blastomeres, the resulting cell-to-cell differences may not cross a functional threshold at the next cell division, but may generate a bias that will subsequently be amplified and become more apparent by the next cleavage stage. This bias will be sufficient to influence cell fate. This viewpoint may reconcile the previously dichotomous debate on the early mammalian embryo being defined as “undifferentiated” or “differentiated”, by emphasizing a “biased” yet “flexible” state.

**Fig. 3:**
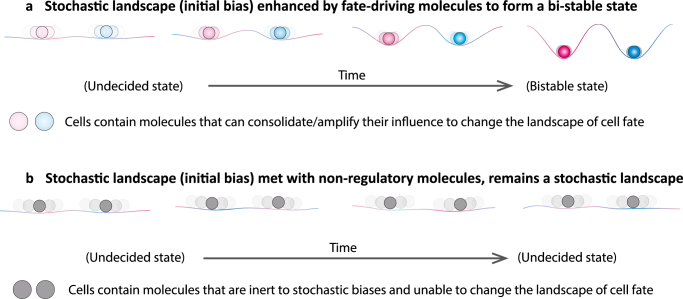
The conditions under which stochastic events may, or may not, drive determinism. The key to whether any initially small bias in molecular expression will either be transformed into a stronger bias or neutralized will depend on the property of the molecule itself. **a** The transformation of a small bias into a strongly defined molecular pattern (bi-stable) would only apply to a subset of molecules (lineage specifiers) that bear the potential to trigger downstream events that are able to consolidate/amplify their influence (enhancing its color) to change the landscape of cell fate. **b** Otherwise, initially small biases will be diluted or reversed by subsequent stochastic fluctuations during development. Our hypothesis represents a view that differs from the classic “Waddington’s landscape” in which the valley, representing the path of a cell lineage, is already set. In our view, the landscape of cell fate could be gradually shaped by molecules that have the potential to “dig” and so alter the landscape as shown in **a**. These principles may apply to both embryonic as well as stem cell fate decisions

Starting with an initially small bias to the transcriptome between blastomeres of 2-cell stage embryos, the subsequent activation of the zygotic genome could trigger further gene regulatory networks that either neutralize or amplify the initial bias^21^. We reason that whether an initially small bias will be transformed into a stronger deterministic-like bias (a “bistable” pattern) or neutralized (a “monostable” pattern) depends on the property of the molecule itself. The transformation of a small bias into a strongly defined molecular pattern would apply to a subset of molecules having the potential to trigger downstream cell fate-driving effects, such as lineage regulators (Fig. 3a). On the other hand, a similar initial heterogeneity in the distribution of “inert molecules” might be quickly diluted or reversed by subsequent stochastic fluctuations during development (Fig. 3b).

### Subcellular behavior of lineage regulators

The expression level of a lineage regulator is a well-known and often discussed factor determining cell fate. However, the subcellular behavior of lineage regulators, meaning their polarized distribution, nuclear accessibility, DNA/RNA-binding ability, and competition/cooperation with other lineage specifiers^84,85^, can be equally powerful.

#### Location matters

**Fig. 4:**
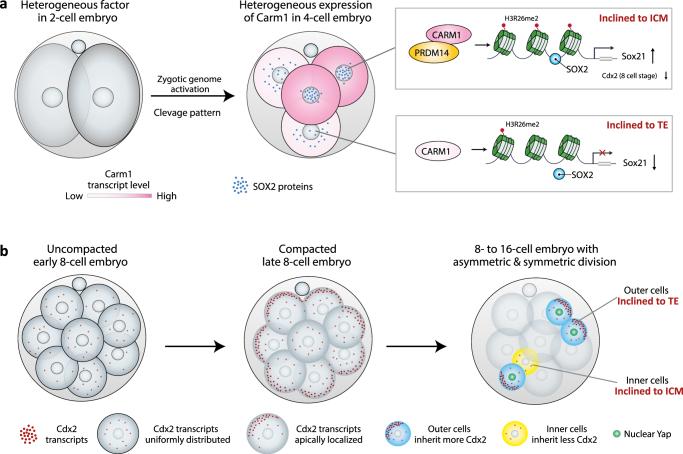
Subcellular compartmentalization of lineage specifiers are key factors for cell fate decisions. **a** Differences between the nuclear location/accessibility of Sox2 at the 4-cell stage embryo are regulated by CARM1, which regulates the level of histone H3R26 methylation^10,11^. This differential behavior of Sox2 leads to differential expression of lineage specifiers such as Sox21, the level of which then directs cell fate as Sox21 is repressor of Cdx2 that directed cell differentiation and is in the positive feedback loop with cell polarization^7, 11^. The identity of a potential factor present at the 2-cell embryo stage that may regulate heterogeneity in CARM1 activity at the 4-cell stage remains unknown. **b** Apically localized Cdx2 transcripts at the late 8-cell stage facilitate the asymmetric distribution of Cdx2 transcripts in daughter cells upon the 8–16 cell division, generating a bias in Cdx2 expression^88^, which segregates cell fate which is further enhanced by nuclear localization of YAP^74–76^

Recent studies have revealed that heterogeneity in the nuclear localization/accessibility (representing the DNA-binding activity) of Sox2 and Oct4 among blastomeres of the 4-cell stage embryo^9,11^ drives cell fate segregation. A blastomere with a long-lived Sox2 or Oct4 DNA binding has been shown to be biased toward forming pluripotent embryonic lineage (ICM), whereas a blastomere with short-lived DNA binding of these transcription factors is biased toward differentiation into the extra-embryonic TE^9,11^ (Fig. 4a). Moreover, the DNA-binding ability of Sox2 is regulated by the extent of the histone H3R26me2 methylation, in turn mediated by the methyltransferase CARM1^11^. Carm1 has also been shown to interact with Prdm14, another epigenetic modifier heterogeneously expressed at the 4-cell stage that biases cell fate toward the ICM when overexpressed^36^. Furthermore, Sox21, a downstream effector of Oct4 and Sox2 also regulated by CARM1, is also heterogeneously expressed at the 4-cell stage^7^ (Fig. 4a). This heterogeneity reflects the orientation and order of the previous cleavage division pattern and is predictive of ICM vs. TE cell fate^7^. These new insights provide molecular evidence of how the intertwined consequences of epigenetic modifications, DNA binding, and a transcriptional cascade, mediated by a group of cooperative lineage regulators, can eventually guide cell fate determination.

At the 8-cell stage, when the embryo develops its apical-basal polarization and compacts, the mRNA for Cdx2, a lineage specifier that drives differentiation into the TE^37,86,87^, has been shown to be asymmetrically localized to the apical pole^37,88^ (Fig. 4b). The asymmetric localization of Cdx2 mRNA requires cell polarization as well as functional microtubule and actin cytoskeletons^88^, which could be highly relevant to the changes in cell shape and thus sub-cellular architecture during this period. The apical localization of Cdx2 transcripts might lead to their differential inheritance upon the next division to the 16-cell stage such that outer cells inheriting the apical domain would have more Cdx2 transcripts than inner cells (Fig. 4b). This would help diverge outer and inner cell fate into the TE and ICM, respectively. Consistent with this concept, daughter cells that inherit the apical domain adopt a TE fate^89,90^. These studies highlight the importance of considering the positional information of lineage specifiers in guiding cell fate in addition to a simple consideration of their expression levels.

#### Competitive switch of cell fates

In addition to differential spatial arrangements of subcellular components providing a potential source of bias, differential fate can also arise in situations where a pair of lineage specifiers with opposing effects co-exists in a single cell, as found in mouse^2,21^ and human^21,91^ embryos from the 8-cell stage onwards. In general, cell fate switching might occur when two opposing lineage specifiers compete for a single shared binding site such as a DNA sequence motif, or a RNA or protein substrate^84,92^. Drosophila ovarian germline stem cells where competitive protein–protein interactions between Bam and the COP9 complex set the cell fate switch between self-renewal or differentiation^92^ provides excellent evidence for this. In such cases, the binding kinetics and thus the wax-and-wane of competition between the determining factors may not only depend on the copy number of lineage specifiers, but also on their intrinsic binding properties, sub-cellular location, and their geographical accessibility to one or more specific sites.

## Conclusions and perspectives

It is an exciting challenge to identify when and how molecular and morphological symmetry breaking emerges and how these two elements integrate spatially and temporally to control the genetic and epigenetic networks that direct lineage fate and pattern formation. We believe that the quantitative integration of both molecular and physical parameters is essential to provide a framework upon which embryonic patterning can be addressed at a biological systems level. Systems-based approaches may permit the development of ways to identify molecules with the potential to guide cell fate based on the properties of known lineage specifiers. Progress in this direction could be made by mining single-blastomere transcriptome data in different mammalian species, using the known properties of lineage specifiers as points of reference. This would be analogous to the use of computational frameworks such as CellNet and Mogrify (network biology-based computational algorithm) designed to predict transcription factors that can most efficiently change cellular state during cellular engineering/reprogramming^93,94^.

There is no doubt that spatial revelation of the compartmentalized intracellular transcriptome and proteome, together with an elucidation of the dynamic behavior of key regulators, will be necessary to fully understand pattern formation in the mammalian embryo, the principles of which could be applicable in stem cell biology. It would also help define molecular and physical circumstances whereby distinct stem cell types can self-organize to form tissues^95^, organoids^96^, embryoids^97–99^, or even artificial embryos made in vitro from stem cells ^100^. Some of these might be distant goals but they present tantalizing possibilities that could push the boundaries of biomedical research.
